# Editorial: Modulation of the immune system by nanoparticles

**DOI:** 10.3389/fimmu.2023.1190966

**Published:** 2023-04-11

**Authors:** Rachel E Hewitt, Mauricio César De Marzi, Kee Woei Ng

**Affiliations:** ^1^ Department of Veterinary Medicine, University of Cambridge, Cambridge, United Kingdom; ^2^ Institute of Ecology and Sustainable Development, National University of Luján, Luján, Argentina; ^3^ School of Materials Science & Engineering, Nanyang Technological University, Singapore, Singapore

**Keywords:** immune modulation, nanoparticles, nanomaterials, extracellular vesicles (EVs), therapeutics, immunotoxicity

The immune system serves as a first line of defense against small materials such as nanoparticles and engineered nanomaterials, but is also open to manipulation by these ultra-small substances. Very small sized materials can interact more efficiently and on a cellular and subcellular level (**Figure 1**), potentially offering a whole new avenue of therapeutic exploration (5, 6). Conversely, these same attributes of very small particles also carry the potential to fuel unwanted immune responses, this phenomenon best described in the field of particulate air pollution (7). Health implications of human exposure to nanoparticles need to be considered in tandem with immune responsiveness. While the principles and approaches to do this are outlined elsewhere (8), this Research Topic collection serves to report some recent developments and provides examples where immune system modulation by nanoparticles is central to the outcomes of the intended applications.

**Figure 1:**
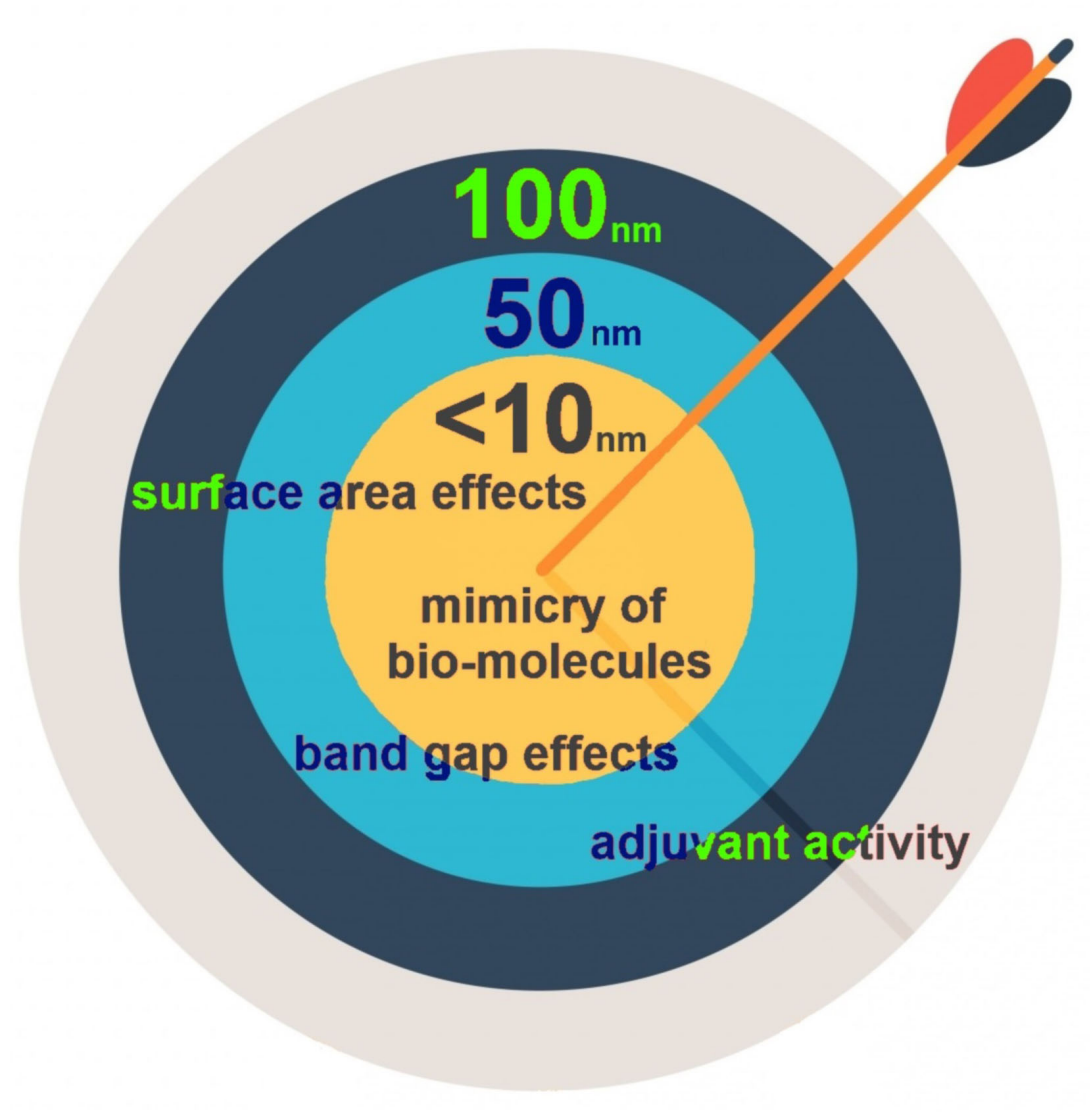
Factors influencing cellular interactions. Nanoparticles within the conventionally defined 1-100 nm size range have the potential to exert a wide range of immuno-suppressive and immune-stimulatory effects, with size, shape, charge, and surface composition all acting to influence the outcome. Particles under 10 nm in diameter are able to interact directly with proteins that trigger biological responses, such as receptors (1, 2). Enhanced reactivity of very small particles results from having large surface areas and increased band gaps in their electronic configurations (3). Particles larger still are effective carriers of antigen or other biologically active cargo to immune cells (4).

Studies on the potential immunotoxic effects of nanoparticles remain very limited. For example, copper oxide nanoparticles (CuO NP) are widely used in industry. Although their potential application as an antimicrobial has been reported, their toxicity is still not well established. In this sense, Tulinska et al. evaluated, in an original article, the effects of CuO NPs on the immune/inflammatory response and antioxidant defense, observing that sub-chronic inhalation of CuO NPs can cause undesired modulation of the immune response. Other commonly used products that include nanoparticles are pigments. An original article by Devcic et al. investigated different cobalt and zinc based pigments used in tattoos and their potential toxic effects on macrophages. All pigments induced functional effects on macrophages (impaired phagocytic capacity, increased secretion of cytokines), which, in some cases, persist long after exposure even at non-toxic doses.

In view of harnessing the immunogenic potential of specific nanoparticles, Chen et al. have compiled a concise review of the immunotherapeutic strategies involving gold nanoparticle based platforms targeting breast cancers. In this sense, Hrvat et al., studied calcium phosphate nanoparticles (CaP-NP) for the delivery of therapeutic molecules in cancer therapies. This original work investigated the effects of CaP-NP and CaP-NP transporting therapeutic antibodies on natural killer (NK) cell activation in ovarian cancer cell line models. The review by Chen et al. provides an in depth view of the current research into the immune-regulatory effects of Selenium nanoparticles and their immunotherapeutic potential. As well as providing an insightful view into the current limitations and challenges in the successful application of Selenium nanoparticles, many of which apply to the therapeutic use of nanoparticles in general. The use of Bilirubin nanoparticles to diminish initial tissue damage in graft versus host responses in a murine mis-matched transplantation model is described in the original research by Pareek et al.

Small extracellular vesicles [EV’s] are another form of small particles with particular relevance in terms of their ability to modulate immune responses. EV’s are lipid-bilayer particles that are released naturally by almost all cells and function to mediate intercellular communication *via* the bioactive molecules contained within and on their surface, such as lipids, proteins, RNA and DNA fragments (9). The bioactive messages and the cellular outcomes of communication *via* EV’s are majorly dependent on the donor cell which produces the EV’s. Mesenchymal stem cells (MSC’s)have been shown to modulate various innate and adaptive immune cells and the clinical applications of MSC-EV’s in cell free liver therapies is reviewed by Wu et al. An original research article by Thome et al. demonstrates the use of regulatory T cell derived EV’s in the suppression of pro-inflammatory responses in an inflammatory mouse model.

This Research Topic aimed to further build on our current understanding of the interactions between nanoparticles and components of the immune system, the consequences of these interactions and how this interplay could be exploited. As the therapeutic application and use of nanoparticles continues to expand, research dedicated to examining the effects of the interactions between nanoparticles, especially ultra-small particles, and the immune system continues to gain importance. Greater knowledge is required to better understand the consequences of environmental exposures, especially in the long term, and importantly to assure the development of safe and effective applications based on nanomaterials.

## Author contributions

All authors listed have made a substantial, direct, and intellectual contribution to the work and approved it for publication.
